# Plant Hormone Response to Low-Temperature Stress in Cold-Tolerant and Cold-Sensitive Varieties of *Zanthoxylum bungeanum* Maxim

**DOI:** 10.3389/fpls.2022.847202

**Published:** 2022-04-29

**Authors:** Jieyun Tian, Yao Ma, Yabing Chen, Xue Chen, Anzhi Wei

**Affiliations:** ^1^College of Forestry, Northwest A&F University, Xianyang, China; ^2^School of Agricultural Sciences, Zhengzhou University, Zhengzhou, China

**Keywords:** *Zanthoxylum bungeanum*, cold stress, plant hormone, chemometric analysis, WGCNA

## Abstract

Plant growth and survival in nature, its growth process, will be affected by various factors from the environment, among which temperature has a greater impact. In recent years, extreme weather has frequently appeared, and the growth of crops has been increasingly affected by the environment. As an important flavoring and Chinese herbal medicine crop, *Zanthoxylum bungeanum* is also facing the harm of low-temperature stress. Plant hormones play a vital role in the response of plants to low temperatures. In this study, ultra-performance liquid chromatography-tandem mass spectrometry (LC-MS/MS) was used to determine the hormone components of cold-tolerant and cold-sensitive varieties of *Z. bungeanum*. Combined with chemometric analysis and weighted gene co-expression network analysis (WGCNA), the hormone component differences and hormone response strategies of *Z. bungeanum* under low-temperature stress were comprehensively studied. The results showed that 45 hormones were detected in *Z. bungeanum*. Among them, there were 7 kinds of components with high content and were detected in both two varieties. At the late stage of low-temperature stress, the contents of abscisic acid (ABA) and ABA-glucosyl ester (ABA-GE) in Fuguhuajiao (FG) were significantly increased, and the latter served as the storage of the former to supplement the active ABA. Orthogonal partial least squares discriminant analysis (OPLS-DA) found that indole-3-carboxylic acid (ICA), indole-3-carboxaldehyde (ICAld), meta-Topolin riboside (mTR), cis-Zeatin-O-glucoside riboside (cZROG), and N6-isopentenyladenosine (IPR) in FG were the upregulated important difference components, and IPR and 2-methylthio-cis-zeatin riboside (2MeScZR) in Fengxiandahongpao (FX) were the upregulated important difference components. There were common crossing points and independent response pathways in response to low temperature in two varieties. WGCNA analysis found that the main hormone components were associated with multiple metabolic pathways including carbon, fatty acid, amino acid, and sugar metabolism, indicating that hormone regulation plays an important role in the response of *Z. bungeanum* to low temperature. This study clarified the hormone response mechanism of *Z. bungeanum* under low-temperature stress and provided a reference and basis for further improving the cold resistance of *Z. bungeanum* and cultivating new varieties.

## Introduction

*Zanthoxylum bungeanum* is a shrub plant of Rutaceae, which is distributed in many Asian countries and has abundant resources in China (Feng et al., 2020). The pericarp of *Z. bungeanum* has become an irreplaceable condiment because of its unique numb flavor, especially in the popular Sichuan hot pot. In addition, the pericarps and leaves can be used in traditional Chinese medicine to treat arthritis, hyperlipidemia, thrombosis, and other diseases, which have a long history in China (Yang et al., 2014; Lei et al., 2016; Alam et al., 2018). At present, the genome of *Z. bungeanum* has been sequenced, further research will be carried out on the numb taste and aroma characteristics (Feng et al., 2021). However, in practical cultivation and production, *Z. bungeanum* plants are often affected by a low-temperature environment, resulting in damage to buds, leaves, and flowers. There are still few studies on the resistance of *Z. bungeanum*, and the response mechanism of low temperature is not clear, which is not conducive to the cultivation of excellent varieties.

To adapt to the adverse low-temperature environment, plants have gradually evolved complex mechanisms to avoid and tolerate cold stress. Among them, the hormone system is an important strategy for plants to deal with low-temperature stress. Many studies have shown that plant hormones play important roles in the low-temperature response. Abscisic acid (ABA) is the most studied and can affect plant growth and development by regulating plant signals. One study showed that the ABA content of *Carpobrotus edulis* increased under cold stress (Fenollosa et al., 2018). Exogenous application of ABA can increase the freezing tolerance of grapevines (Wang et al., 2020). Under low-temperature stress, the expression pathways of response genes in plants were divided into ABA-dependent and ABA-independent pathways, which depended on ABA/AREB and DREB/CBF. Auxin is mainly involved in organogenesis and morphogenesis, which are related to the accumulation and polarity distribution of auxin in plant tissues (Rahman, 2013). Shibasaki confirmed that cold stress mainly affected the transport of auxin in cells, and cold blocked the asymmetric distribution of auxin efflux carrier component 3 (PIN3) (Shibasaki et al., 2009). Low temperature can inhibit the gravitropism of auxin in plants. Therefore, the intracellular auxin response is related to the developmental regulation of plant growth under cold stress. As an important growth regulator regulating plant physiological and biochemical characteristics under abiotic stress, salicylic acid (SA) can reduce stress damage by increasing the responses of proline, antioxidants, heat shock protein, secondary metabolism, and sugar, thus improving the tolerance of plants to stress (Ahmad et al., 2019; Arif et al., 2020). It was found that low-temperature stress increased the content of endogenous SA in cucumber, and the expression of COR genes in grafted cucumber (*Cucumis sativus* L.) was significantly upregulated after the application of exogenous SA (Fu et al., 2021). Other hormones such as ethylene have also been reported to enhance cold resistance by increasing the activity of the antioxidant enzyme system (Ohme-Takagi and Shinshi, 1995). Cytokinin response factors *CRF2* and *CRF3* have been reported to regulate lateral root development in *Arabidopsis thaliana* under low-temperature stress (Jeon et al., 2016). Jasmonic acid (JA) was found to be a key upstream signal of the ICE-CBF/DREB1 pathway positively regulating cold tolerance in *A. thaliana* (Hu et al., 2013). Lange found that the gene encoding gibberellin-related catabolic enzyme *AtGA2ox9* increased the transcriptional level after low-temperature treatment in *A. thaliana* and helped to improve cold tolerance (Lange et al., 2020). The hormone response of plants under low-temperature stress is not regulated by a single hormone, but coordinated or antagonistic. In this way, the dynamic balance network of the plant hormone system is formed, which effectively helps plants maintain normal growth and development requirements under stress. At present, studies on hormone metabolism and regulation of *Z. bungeanum* under low-temperature stress have not been reported, and the co-regulation strategy of multiple hormone components in *Z. bungeanum* is not clear.

In this study, cold-tolerant and cold-sensitive varieties of *Z. bungeanum* were selected, and the hormone components were determined by liquid chromatography-tandem mass spectrometry (LC-MS/MS). Chemometric analysis was employed to analyze the differences and changes in hormone components of *Z. bungeanum* under low temperatures. Using weighted co-expression network analysis (WGCNA) combined with hormone component content and transcriptome sequencing analysis, we explored the pathways and genes related to hormone responses of *Z. bungeanum* under cold stress. The hormone response characteristics and strategies of two *Z. bungeanum* varieties under low-temperature stress were investigated at the metabolome and transcriptome levels, which provided the basis for further study on measures to improve the cold resistance of *Z. bungeanum*.

## Materials and Methods

### Plant Materials and Cold Treatment

Two varieties, “Fuguhuajiao” (FG) and “Fengxiandahongpao” (FX) of *Z. bungeanum*, were selected for the study. The variety FG was from the areas with higher latitude and lower temperatures (38°42′ N~39°35′ N, 110°22′ E~111°14′ E, mean temperature 9.1°C). FG plants bloom late in the spring and have strong stress-resistant growth characteristics. The variety FX was from the areas with relatively high temperatures (33°34′ N~34°18′ N, 106°24′ E~107°7′ E, mean temperature 12.1°C). The FX plants germinate earlier in the spring and have cold-sensitive growth characteristics. Seedlings of the cold-tolerant variety “Fuguhuajiao” and the cold-sensitive variety “Fengxiandahongpao” of *Z. bungeanum* were planted in the greenhouse (25–28°C, 16/8 h day/night) of Northwest A&F University. The seeds were collected from the Research Center for Engineering and Technology of *Zanthoxylum*, State Forestry Administration, Northwest A&F University, Fengxian, Shaanxi Province, China. Cold treatments were performed on the 4-month-old seedlings under 4°C for 24 h in a growth chamber. Leaf samples of seedlings were collected after low-temperature stress treatments for 0, 1, 3, 6, 12, and 24 h, and immediately put into liquid nitrogen. The samples were then transferred to the −80°C ultra-low temperature refrigerator for further determination and analysis. “Fuguhuajiao” and “Fengxiandahongpao” were marked as FG (FG1-18, 6 time points ×3 biological replicates) and FX (FX1-18, 6 time points ×3 biological replicates).

### Plant Hormone Extraction and Composition Analysis by UPLC-MS/MS

The plant hormones in ground samples (50 mg) were extracted by the mixed solvent containing methanol/water/formic acid (15:4:1, v/v/v) and the internal standard (100 ng/ml). After 10 min of vortexing, the samples were centrifuged at 4°C, 12,000 r/min, and the supernatant was taken to a new centrifuge tube for concentration. The concentrated samples were redissolved with 100 μl of 80% methanol/water solution, filtered, and placed in the sample injection bottles for ultra-performance liquid chromatography-tandem mass spectrometry (UPLC-MS/MS) analysis. The standard solutions of 0.01, 0.05, 0.1, 0.5, 1, 5, 10, 50, 100, 200, and 500 ng/ml were prepared to obtain the mass spectrum peak intensity data of the corresponding quantitative signal of each concentration standard. The concentration ratio of external standard to internal standard was the abscissa, and the peak area ratio of external standard to internal standard was the ordinate. The standard curves of different substances were drawn.

The UPLC-MS/MS analysis was performed on the Waters Acquity ExionLCTM AD coupled with QTRAP 6500+ LC-MS/MS system (AB Sciex Pte. Ltd). The UPLS HSS T3 C18 column (1.8 μm, 100 mm × 2.1 mm) was equipped for plant hormone determination. The conditions included were as follows: mobile phase A was water with 0.04% acetic acid, mobile phase B was acetonitrile with 0.04% acetic acid; the flow rate was 0.35 ml/min, and the column temperature was 40°C. The gradient elution procedure was as follows: A/B 95:5 (v/v) from 0 to 1 min, A/B 5:95 (v/v) from 8 to 9 min, and A/B 95:5 (v/v) from 9 to 12 min. The quantitative analysis of plant hormone components was completed by using the multiple reaction monitoring (MRM) of a triple quadrupole mass spectrometer.

### Data Analysis

Statistically significant differences were performed by ANOVA using SPSS version 19.0 (IBM Corp., Armonk, NY, USA). Data standardization and the principal component analysis (PCA) were conducted using OriginPro 2017C (Originlab, Northampton, USA). The cluster heat map (CHM) was performed using TBtools (Chen et al., 2020). The orthogonal partial least squares discriminant analysis (OPLS-DA), the uniform manifold approximation and projection (UMAP) and the nonmetric multidimensional scaling (NMDS) were performed by using online software (https://www.omicshare.com/tools/Home/Soft/getsoft).

The WGCNA was carried out using the R package (Langfelder and Horvath, 2008). The genes from transcriptomes of 36 cold-treated leaf samples were used as the input data for co-expression network construction. Transcriptome data for WGCNA were derived from our previous work (Tian et al., 2021). The parameters were power = 15, minModuleSize = 30, and mergeCutHeight = 0.25. The connectivity of hub genes was the sum of the weights from all edges of a node. The co-expression network was visualized by Cytoscape 3.7.1 (Shannon et al., 2003).

## Results

### Identification and Changing Profiles of Plant Hormone Components in Two *Z. bungeanum* Varieties

**Figure 1:**
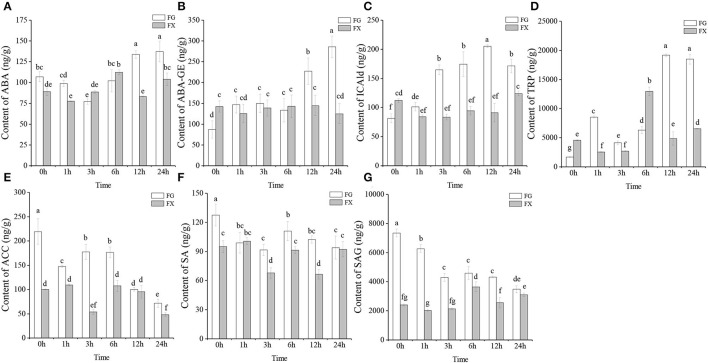
**(A–G)** The main plant hormone components in cold-tolerant variety FG and cold-sensitive variety FX of *Zanthoxylum bungeanum*.

To obtain the plant hormone effect on *Z. bungeanum* under low-temperature stress, UPLC-MS/MS was operated on cold-tolerant and cold-sensitive varieties of leaves for analyzing the plant hormone components. A total of 45 plant hormone components were detected in two varieties of *Z. bungeanum*: 2 ABAs (abscisic acid, ABA; ABA-glucosyl ester, ABA-GE), 15 auxins (3-indoleacrylic acid, IA; indole-3-acetic acid, IAA; N-(3-indolylacetyl)-L-alanine, IAA-Ala; 1-O-indol-3-ylacetylglucose, IAA-Glc; indole-3-acetyl glutamic acid, IAA-Glu; indole-3-acetyl glycine, IAA-Gly; 3-indoleacetonitrile, IAN; indole-3-butyric acid, IBA; indole-3-carboxylic acid, ICA; indole-3-carboxaldehyde, ICAld; indole-3-lactic acid, ILA; methyl indole-3-acetate, MEIAA; 2-oxindole-3-acetic acid, OxIAA; tryptamine, TRA; L-tryptophan, TRP), 17 CKs (*trans*-zeatin, tZ; N6-isopentenyladenine, IP; meta-topolin riboside, mTR; kinetin riboside, KR; N6-isopentenyl-adenine-9-glucoside, iP9G; *cis*-zeatin-O-glucoside riboside, cZROG; *cis*-zeatin riboside, cZR; *cis*-zeatin-9-glucoside, cZ9G; N6-isopentenyladenosine, IPR; kinetin-9-glucoside, K9G; 2-chloro-*trans*-zeatin, 2CltZ; *trans*-zeatin riboside, tZR; dihydrozeatin-O-glucoside riboside, DHZROG; dihydrozeatin ribonucleoside, DHZR; dihydrozeatin-7-glucoside, DHZ7G; 2-methylthio-*cis*-zeatin riboside, 2MeScZR; *trans*-zeatin-O-glucoside, tZOG), 1 ETHs (1-aminocyclopropanecarboxylic acid, ACC), 2 GAs (gibberellin A19, GA19; gibberellin A9, GA9), 5 JAs (jasmonic acid, JA; jasmonoyl-L-isoleucine, JA-ILE; N-[(-)-jasmonoyl]-(L)-valine, JA-Val; dihydrojasmonic acid, H2JA; *cis*(+)-12-oxophytodienoic acid, OPDA), 2 SAs (salicylic acid, SA; salicylic acid 2-O-β-glucoside, SAG), and 1 SLs (5-deoxystrigol, 5DS) (Table 1). Among them, ABA, ABA-GE, ICAld, TRP, ACC, SA, and SAG were detected in the samples of both cold-tolerant and cold-sensitive varieties with high hormone contents (Figures 1A–G).

**Table 1:** Identification of plant hormone components in two varieties of *Zanthoxylum bungeanum*.

**Plant hormone**	**Compound name**	**Class**
ABA	Abscisic acid	ABAs
ABA-GE	ABA-glucosyl ester	ABAs
IA	3-Indoleacrylic acid	Auxins
IAA	Indole-3-acetic acid	Auxins
IAA-Ala	N-(3-Indolylacetyl)-L-alanine	Auxins
IAA-Glc	1-O-indol-3-ylacetylglucose	Auxins
IAA-Glu	Indole-3-acetyl glutamic acid	Auxins
IAA-Gly	Indole-3-acetyl glycine	Auxins
IAN	3-Indoleacetonitrile	Auxins
IBA	Indole-3-butyric acid	Auxins
ICA	Indole-3-carboxylic acid	Auxins
ICAld	Indole-3-carboxaldehyde	Auxins
ILA	Indole-3-lactic acid	Auxins
MEIAA	Methyl indole-3-acetate	Auxins
OxIAA	2-oxindole-3-acetic acid	Auxins
TRA	Tryptamine	Auxins
TRP	L-tryptophan	Auxins
tZ	trans-Zeatin	CKs
IP	N6-isopentenyladenine	CKs
mTR	meta-Topolin riboside	CKs
KR	Kinetin riboside	CKs
iP9G	N6-Isopentenyl-adenine-9-glucoside	CKs
cZROG	cis-Zeatin-O-glucoside riboside	CKs
cZR	cis-Zeatin riboside	CKs
cZ9G	cis-Zeatin-9-glucoside	CKs
IPR	N6-isopentenyladenosine	CKs
K9G	Kinetin-9-glucoside	CKs
2CltZ	2-Chloro-trans-zeatin	CKs
tZR	trans-Zeatin riboside	CKs
DHZROG	Dihydrozeatin-O-glucoside riboside	CKs
DHZR	Dihydrozeatin ribonucleoside	CKs
DHZ7G	Dihydrozeatin-7-glucoside	CKs
2MeScZR	2-Methylthio-cis-zeatin riboside	CKs
tZOG	trans-Zeatin-O-glucoside	CKs
ACC	1-Aminocyclopropanecarboxylic acid	ETHs
GA19	Gibberellin A19	GAs
GA9	Gibberellin A9	GAs
JA	Jasmonic acid	JAs
JA-ILE	Jasmonoyl-L-isoleucine	JAs
JA-Val	N-[(-)-Jasmonoyl]-(L)-valine	JAs
H2JA	Dihydrojasmonic acid	JAs
OPDA	cis(+)-12-Oxophytodienoic acid	JAs
SA	Salicylic acid	SAs
SAG	Salicylic acid 2-O-β-glucoside	SAs
5DS	5-Deoxystrigol	SLs

The changing trends of ABA and ABA-GE in FG and FX under cold stress were different. In FG, the content of ABA decreased from 0 to 3 h, then increased to the peak at 24 h (137.26 ng/g) (Figure 1A). In FX, ABA content basically showed an increasing trend first and then decreased, and reaching the peak value (112.42 ng/g) at 6 h. The content of ABA in FG was significantly higher than that in FX at the early (0–1 h) and late (12–24 h) stage under cold stress. We also noticed that the content of another ABAs component, ABA-GE, increased significantly at the late stage (12–24 h) in FG, and the ABA-GE content at 24 h was 3.29-fold and 1.93-fold compared with that at 0 h in FG and 24 h in FX, respectively (Figure 1B). In FX, the content of ABA-GE increased slightly during low-temperature stress, but the change was not obvious. The content of auxin component ICAld in FG showed a gradually increasing trend, which increased significantly at 3 h and reached the peak (205.29 ng/g) at 12 h under cold stress (Figure 1C). In FX, the content of ICAld was higher than that of FG only at 0 h and lower than FG in the process of low-temperature stress. The content level of TRP, another auxin component, was the highest among *Z. bungeanum*. The content of TRP was low under the normal growth in FG, but increased significantly and reached the peak at 12 h (19,150.33 ng/g) after cold stress, and maintained a significantly high level at 24 h (18,484.77 ng/g) (Figure 1D). In FX, the content of TRP increased to the peak value at 6 h (12,960.14 ng/g), then decreased rapidly. The content of ETH component ACC in *Z. bungeanum* was also at a high level (Figure 1E). In FG, the contents of ACC, SA, and SAG all showed a downward trend, and the contents in the samples treated with low temperature were lower than those in the control (0 h). In addition, the contents of the above three hormones in FG were generally higher than those in FX. On the whole, the 7 plant hormone components with high content in *Z. bungeanum* were higher in FG than in FX at the late stage (12–24 h) of low-temperature stress.

### Chemometric Analysis of Plant Hormone Components in *Z. bungeanum*

As many hormone components were detected in *Z. bungeanum* under low-temperature stress, the content-changing profile of the two varieties under different treatment times needs to be further analyzed. Therefore, using a variety of chemometric analysis methods, including linear and nonlinear analysis models, to analyze the hormone components under cold stress treatment can make the response of plant hormone in *Z. bungeanum* clearer and screen out the important differential hormone components.

#### Cluster Heat Map Analysis

**Figure 2:**
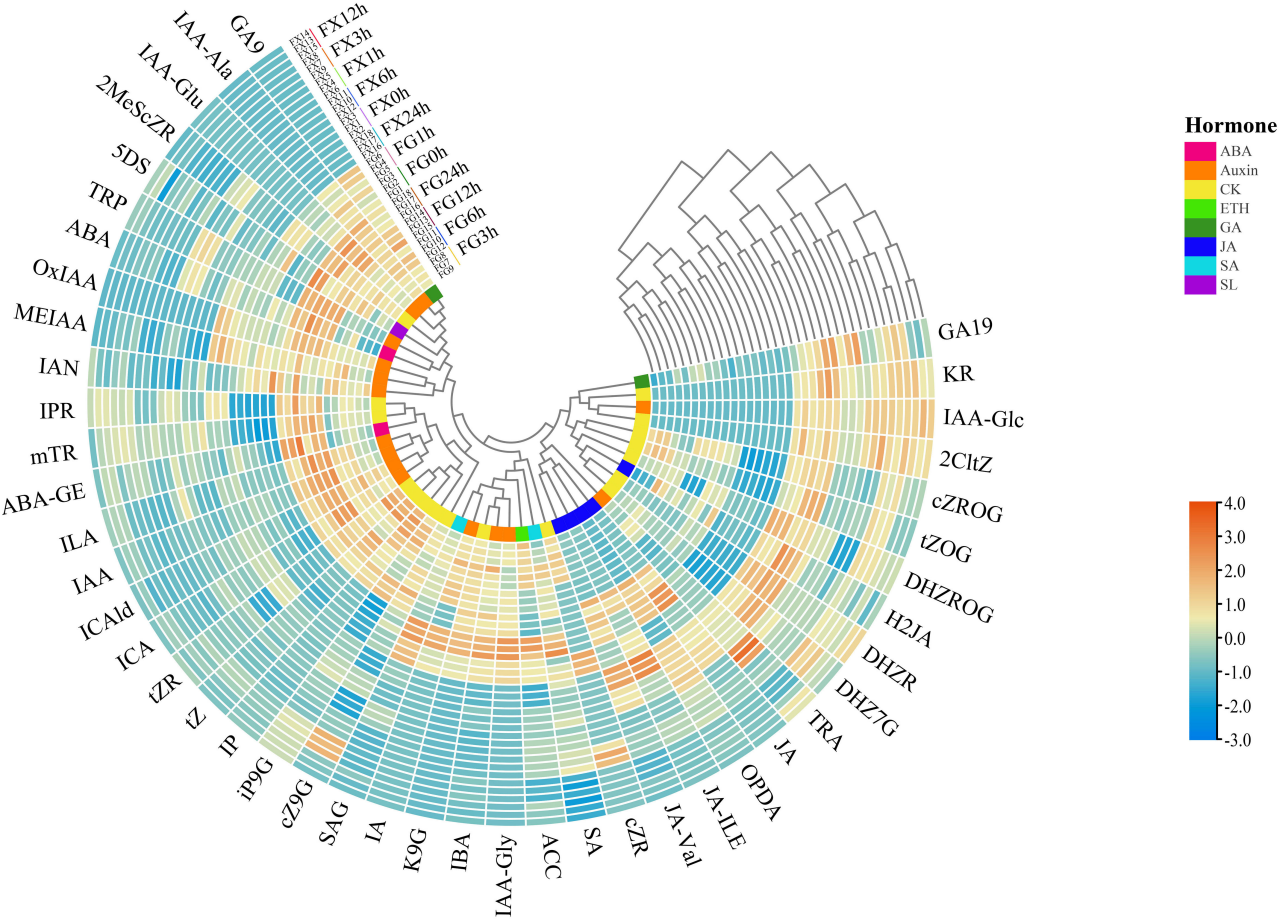
The cluster heat map of the plant hormone components for *Z. bungeanum* varieties FG and FX.

Through the cluster heat map analysis of the samples in FG and FX under low-temperature stress treatment, we can preliminarily understand the classification of different samples due to the influence of hormone components, as well as the classification differences and characteristics. The normalized data were used for cluster analysis, and the results showed that 36 samples of *Z. bungeanum* were divided into four groups: FG7-9 (3 h) and FG10-12 (6 h) were the first group, FG13-15 (12 h) and FG16-18 (24 h) were the second group, FG1-3 (0 h) and FG4-6 (1 h) were the separate third group, tall FX samples FX1-18 (0–24 h) were divided into the fourth group (Figure 2). In addition, the 45 plant hormone components were grouped into four clusters: the first group included two subgroups, 10 hormone components (GA9, IAA-Ala, IAA-Glu, 2MeScZR, 5DS, TRP, ABA, OxIAA, MEIAA, and IAN) were divided into one subgroup, and 12 (IPR, mTR, ABA-GE, ILA, IAA, ICAld, ICA, tZR, tZ, IP, iP9G, and cZ9G) were divided into another subgroup, 8 components (SAG, IA, K9G, IBA, IAA-Gly, ACC, SA, and cZR) were grouped into the second cluster, 5 components (JA-Val, JA-ILE, OPDA, JA, and TRA) were in the third cluster, the fourth group also included two subgroups, 6 components (DHZ7G, DHZR, H2JA, DHZROG, tZOG, and cZROG) were divided into one subgroup, 4 (2CltZ, IAA-Glc, KR, and GA19) were in another subgroup. The same color in the innermost circle in Figure 2 represents the same plant hormone class. It was found that some hormone components in the same class were divided into different groups.

#### Principal Component Analysis

**Figure 3:**
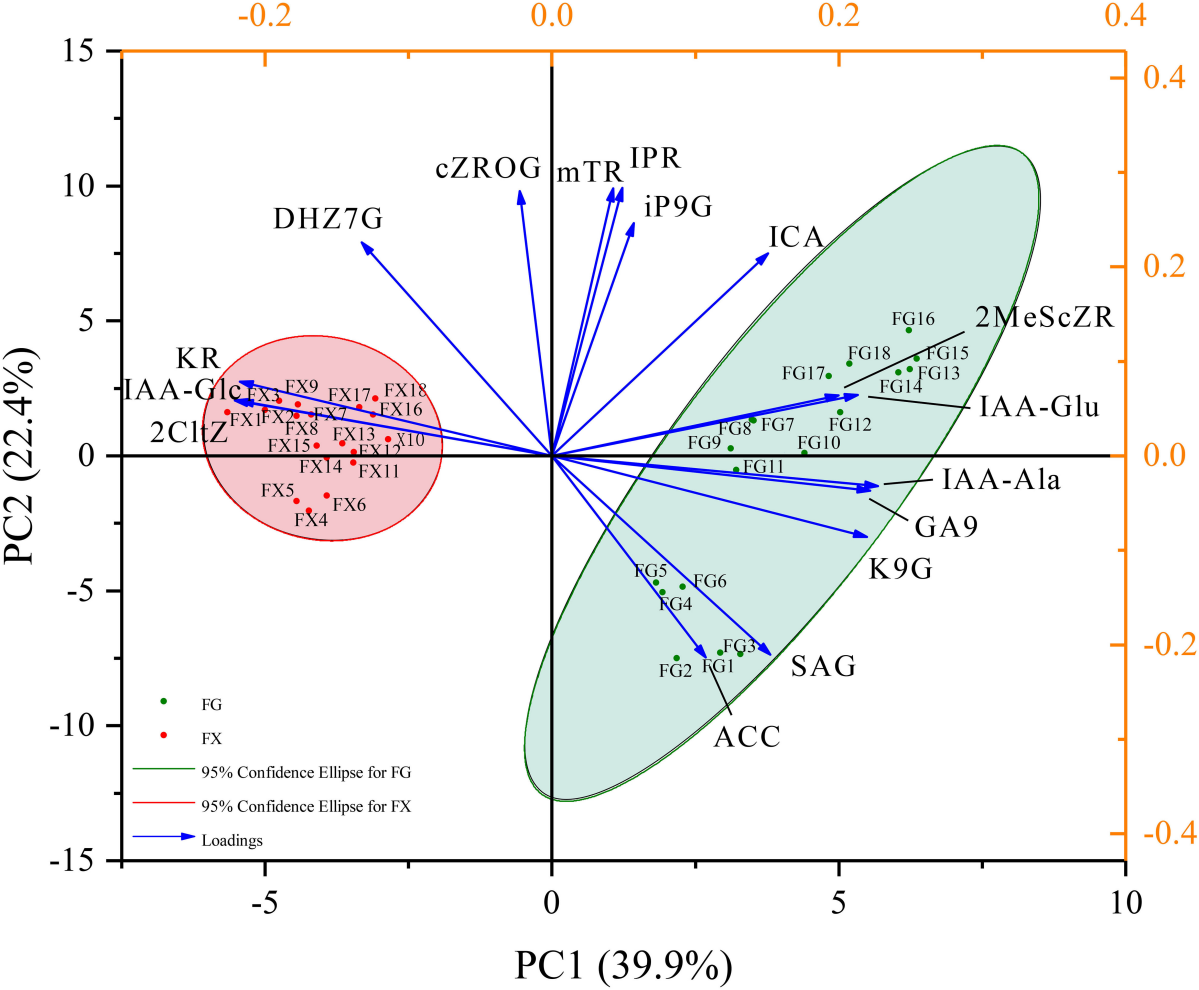
The principal component analysis (PCA) of the plant hormone components for *Z. bungeanum* varieties FG and FX.

Principal component analysis was used to reduce the dimension of hormone components in *Z. bungeanum* under cold stress, and the components with the highest contribution rates were selected. Through the PCA analysis of 45 hormone components in 36 samples of two *Z. bungeanum* varieties, the eigenvalues and cumulative contribution rates of each component were obtained (Table 2). The indicators with an eigenvalue > 1 were selected as the main indicators of principal components. In this result, the eigenvalues of the top eight were >1, with the cumulative contribution rate reaching 92.85%. The results showed that in the first principal component (PC1), IAA-Ala, GA9, K9G, IAA-Glu, and 2MeScZR were the main positive indicators of plant hormone components, and IAA-Glc, 2CltZ, and KR were the main negative indicators (Figure 3). In PC2, IPR, mTR, cZROG, iP9G, DHZ7G, and ICA were the main positive indicators, while SAG and ACC were the negative indicators. In addition, PC1 clearly separated the samples of two *Z. bungeanum* varieties, while PC2 divided the samples into FG and FX treated with cold stress at different time points. Therefore, the 16 hormone components with an important cumulative contribution rate screened by PCA were selected as the main indicators in *Z. bungeanum* for further analysis.

**Table 2:** Eigenvalue and cumulative of plant hormone components.

**Principal component number**	**Eigenvalue**	**Cumulative (%)**
1	17.95	39.88
2	10.09	62.31
3	4.82	73.02
4	2.83	79.29
5	2.03	83.81
6	1.84	87.90
7	1.13	90.40
8	1.11	92.85
9	0.85	94.74
10	0.52	95.91
11	0.47	96.96
12	0.31	97.64
13	0.24	98.19
14	0.16	98.53
15	0.12	98.81
16	0.09	99.02
17	0.07	99.18
18	0.07	99.33
19	0.06	99.47
20	0.04	99.56
21	0.04	99.65
22	0.03	99.72
23	0.03	99.78
24	0.02	99.83
25	0.02	99.87
26	0.01	99.91
27	0.01	99.93
28	0.01	99.95
29	0.01	99.96
30	0.01	99.98
31	0.00	99.98
32	0.00	99.99
33	0.00	100.00
34	0.00	100.00
35	0.00	100.00
36	0.00	100.00

#### Uniform Manifold Approximation and Projection Analysis

Furthermore, we took the 7 main hormone components with high content and detected in both two varieties and 16 hormone components with important contribution rates screened by PCA as the main hormone indicators for further analysis, including ABA, ABA-GE, IAA-Ala, IAA-Glc, IAA-Glu, ICA, ICAld, TRP, mTR, KR, iP9G, cZROG, IPR, K9G, 2CltZ, DHZ7G, 2MeScZR, ACC, GA9, SA, and SAG.

**Figure 4:**
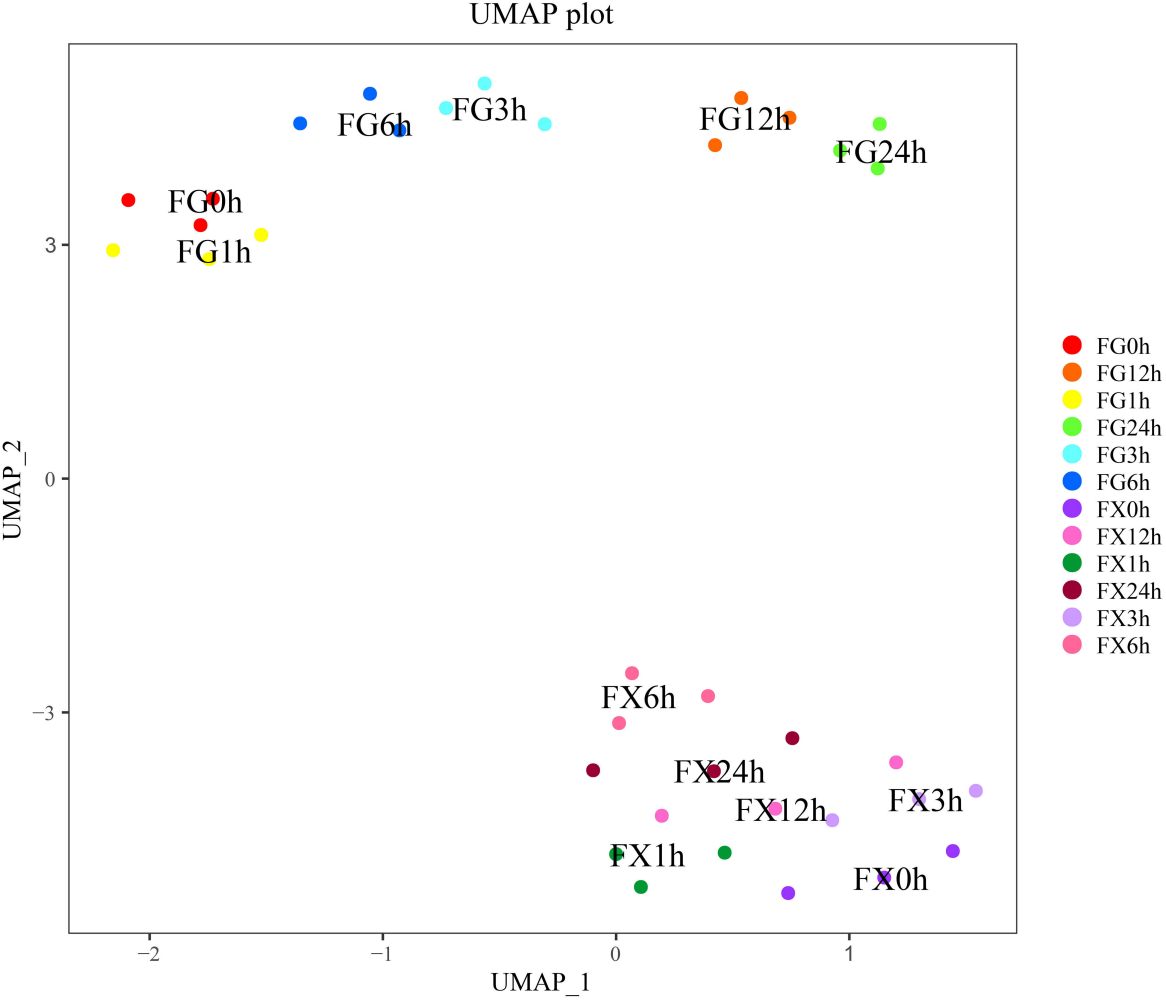
The uniform manifold approximation and projection (UMAP) of the plant hormone components for *Z. bungeanum* varieties FG and FX.

The nonlinear analysis model uniform manifold approximation and projection analysis (UMAP) was carried out to analyze the above 21 hormone components and project them to a two-dimensional coordinate system to visualize their distribution. The results showed that the distance between FG1-3 (0 h) and FG4-6 (1 h) was close, FG7-9 (3 h) and FG10-12 (6 h) was close, and FG13-15 (12 h) and FG16-18 (24 h) was close (Figure 4). This result was consistent with the result of cluster analysis, indicating that the above 21 hormone component indicators can represent the overall hormone response level in *Z. bungeanum* samples, and the data distribution was also conformed to the nonlinear model, which can be explained and analyzed by the nonlinear model.

#### Nonmetric Multidimensional Scaling Analysis

**Figure 5:**
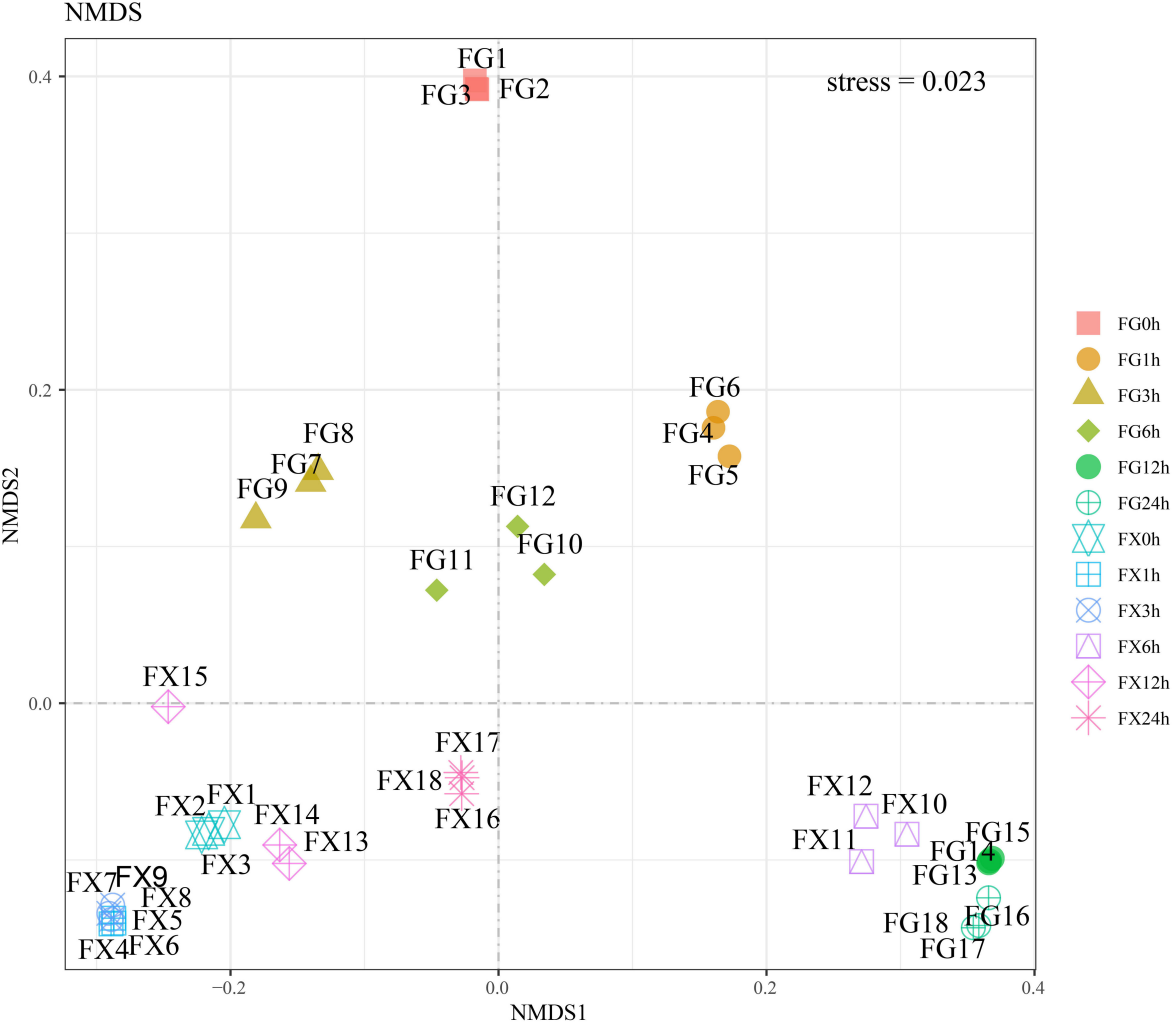
The nonmetric multidimensional scaling (NMDS) of the plant hormone components for *Z. bungeanum* varieties FG and FX.

Nonmetric multidimensional scaling analysis was used to analyze 21 main plant hormone components in cold-tolerant and cold-sensitive varieties of *Z. bungeanum* samples under cold stress. The results showed that the stress value was 0.023 (<0.05), indicating that the model had good representativeness and the model accurately reflected the real distribution of data sorting (Figure 5). NMDS analysis showed that the samples of FG and FX treated with low-temperature stress were obviously separated, and the samples at different treatment time points had certain differences. The plant hormone levels and components of FG in a normal growth state were significantly different from those of other samples. The hormone components in the samples of FX10-12 (6 h) to FG13-15 (12 h) and FG16-18 (24 h) were relatively close.

#### Orthogonal Partial Least Square Discriminant Analysis

**Figure 6:**
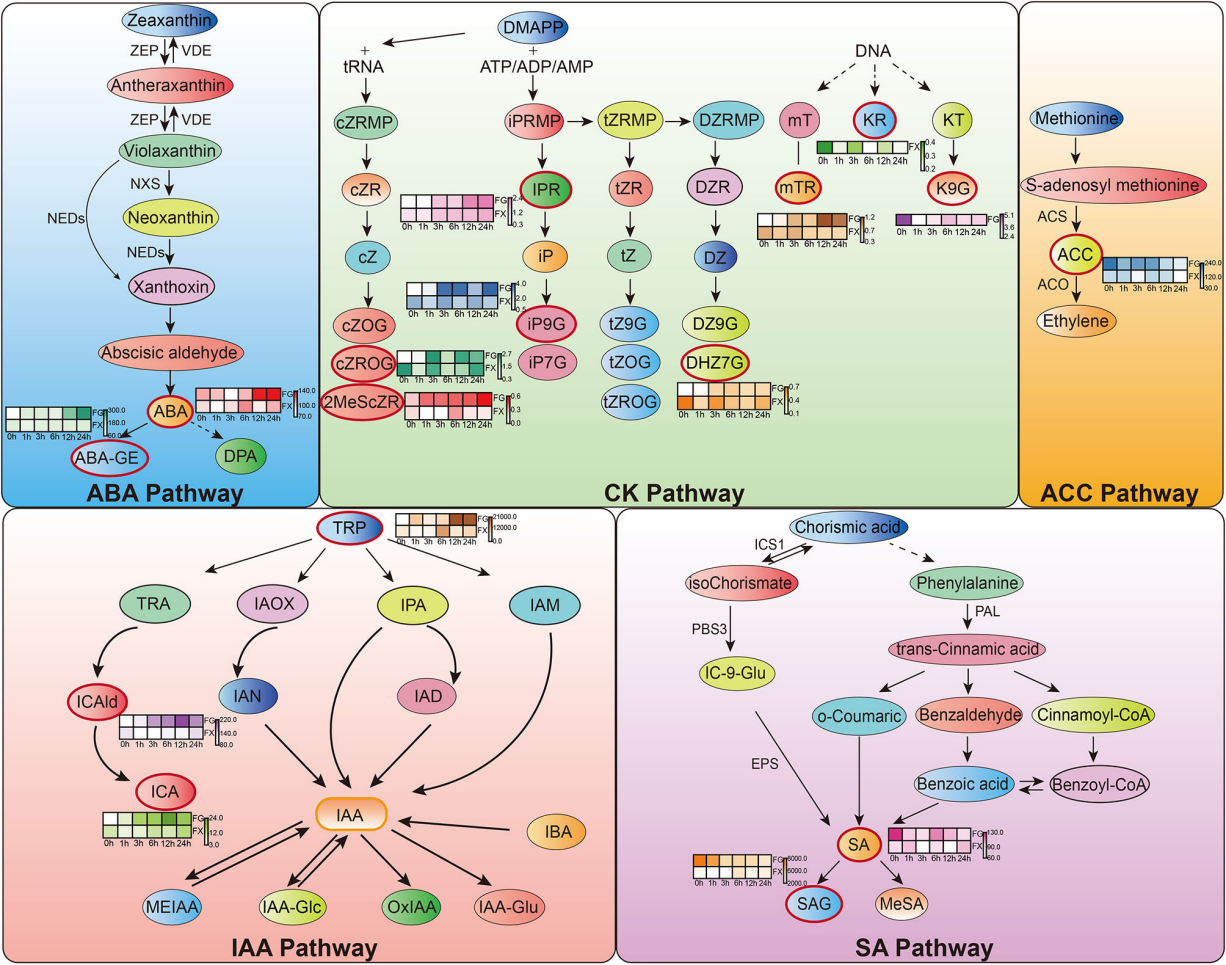
Hormone response pathways in *Z. bungeanum* under cold stress. Red boxes indicate important hormone components identified from orthogonal partial least squares discriminant analysis (OPLS-DA). The heat map graph beside them shows the content change pattern of each important hormone component.

Orthogonal partial least square discriminant analysis was used to determine the differences in hormone components in two varieties and to identify the important differential hormone substances under cold stress. Variable important in projection (VIP) threshold was introduced to measure the influence intensity of different hormone components in low temperature-treated samples. In this study, plant hormone components with VIP > 1 were considered to have a strong influence and explanatory power on the cold stress treatment sample (Table 3). OPLS-DA plot showed a clear separation of the two varieties. Results showed that in FG, 15 hormone components, namely, ABA, ABA-GE, ICA, ICAld, TRP, mTR, iP9G, cZROG, IPR, K9G, DHZ7G, 2MeScZR, ACC, SA, and SAG, were important feature hormone metabolites during the 24 h process of cold stress. Among them, the key hormone components from 3 h of cold stress were ICA, ICAld, mTR, cZROG, and IPR, and all of them were upregulated differential substances. There were also 15 important differential substances in FX, which were ABA, ICA, ICAld, TRP, mTR, KR, iP9G, cZROG, IPR, 2CltZ, DHZ7G, 2MeScZR, ACC, SA, and SAG. Among them, KR and DHZ7G were the key differential hormone components in the process of low-temperature stress and showed a downregulated pattern. We noticed that the pattern of IPR in FX was consistent with FG, both of which were important differentially upregulated hormone components from 3 to 24 h. The results indicated that *Z. bungeanum* mainly responded to low temperature through the important hormone components in the ABA pathway, CK pathway, ACC pathway, IAA pathway, and SA pathway (Figure 6).

**Table 3:** Variable important for the projection (VIP) of plant hormone components in orthogonal partial least squares discriminant analysis (OPLS-DA).

	**FG1h**	**FG3h**	**FG6h**	**FG12h**	**FG24h**	**FX1h**	**FX3h**	**FX6h**	**FX12h**	**FX24h**
ABA	0.68	1.02	0.23	0.81	0.82	0.94	0.05	1.26	0.68	1.15
ABA-GE	1.25	0.84	0.74	1.13	1.33	0.53	0.13	0.01	0.08	0.55
IAA-Ala	0.83	0.38	0.20	0.03	0.31	0.00	0.00	0.00	0.00	0.00
IAA-Glc	0.00	0.00	0.00	0.00	0.00	0.71	0.43	0.97	0.77	0.25
IAA-Glu	0.85	0.01	0.13	0.79	0.83	0.00	0.00	0.00	0.00	0.00
ICA	0.94	1.14	1.36	1.36	1.05	0.95	0.98	0.30	0.09	1.06
ICAld	0.79	1.18	1.37	1.22	1.01	1.04	1.30	0.71	0.90	0.72
TRP	1.37	0.55	0.83	1.24	1.19	0.76	0.90	1.40	0.16	0.83
mTR	0.77	1.27	1.27	1.36	1.23	0.68	0.12	0.78	0.97	1.05
KR	0.00	0.00	0.00	0.00	0.00	1.39	1.11	1.35	1.31	1.53
iP9G	0.76	1.47	1.62	0.94	1.26	1.17	0.43	0.85	0.02	0.96
cZROG	0.66	1.47	1.13	1.18	1.07	1.76	0.93	1.44	1.64	0.64
IPR	0.19	1.17	1.21	1.23	1.23	0.49	1.35	1.05	1.34	1.57
K9G	1.35	0.80	0.62	0.62	0.55	0.00	0.00	0.00	0.00	0.00
2CltZ	0.00	0.00	0.00	0.00	0.00	0.49	0.02	1.03	0.40	0.07
DHZ7G	0.44	1.09	1.28	0.85	0.87	1.84	1.10	1.40	1.96	1.49
2MeScZR	1.37	0.90	1.04	0.83	1.02	1.03	1.23	1.17	0.64	0.72
ACC	1.44	0.66	0.74	1.07	1.17	0.54	1.50	0.29	0.25	1.43
GA9	0.60	0.02	0.50	0.63	0.02	0.00	0.00	0.00	0.00	0.00
SA	1.49	1.19	0.78	0.82	0.92	0.55	1.96	0.35	1.86	0.25
SAG	0.99	1.16	1.20	0.97	1.08	0.62	0.62	1.01	0.28	0.93

### Analysis of the Co-Expression Network of Important Differential Hormones and Transcriptome in Response to Low Temperature in *Z. bungeanum*

**Figure 7:**
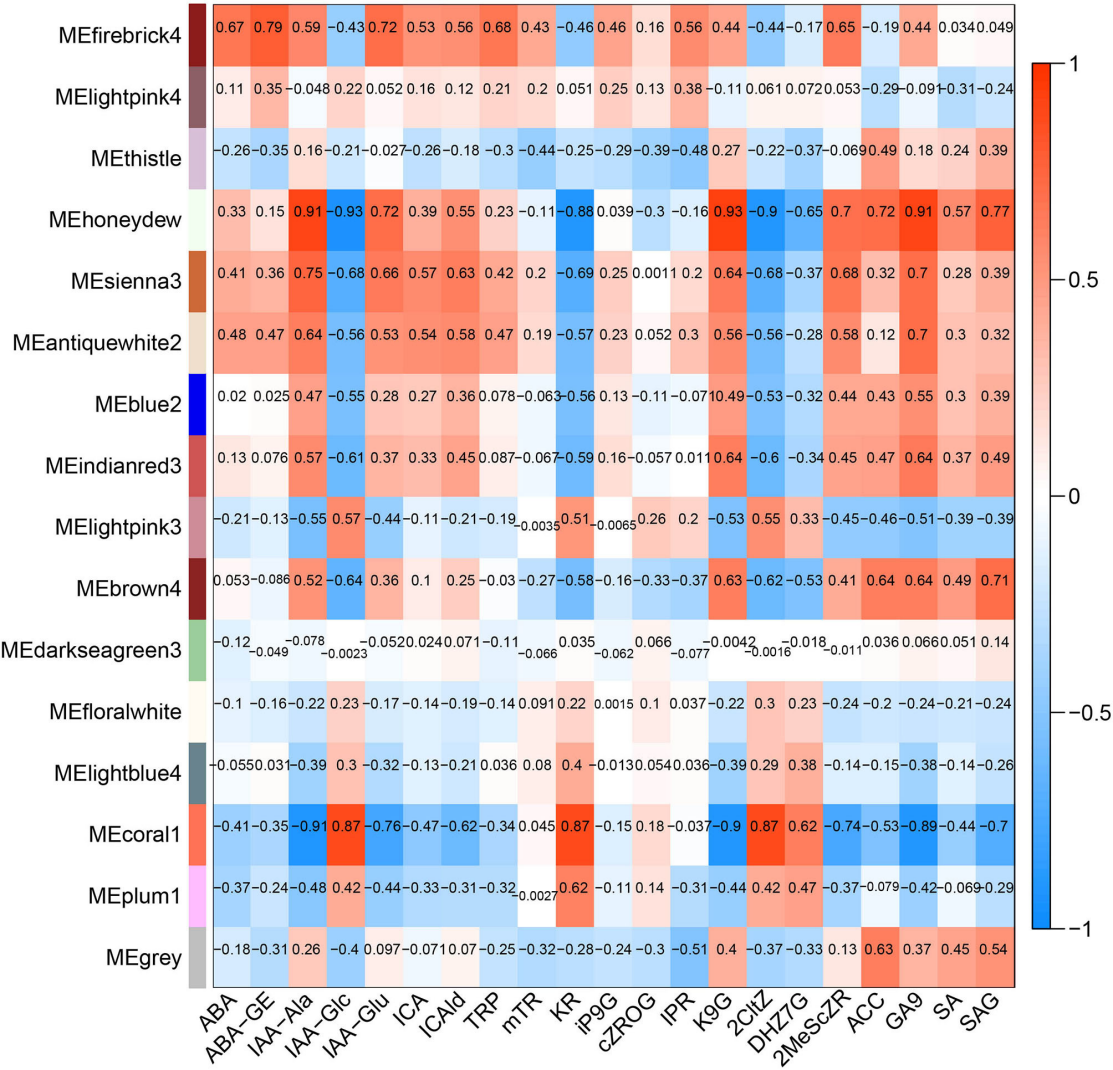
Correlations between modules and dominating plant hormone component traits in *Z. bungeanum*.

The hormone-related gene network in *Z. bungeanum* was constructed by combining 12 main hormone components with a transcriptome profile by WGCNA. After optimizing and merging the imported data, 16 modules were finally obtained. The results showed that several modules had a high correlation with hormone components (Figure 7). Among them, the MEhoneydew module had a high correlation with IAA-Ala, IAA-Glc, IAA-Glu, KR, K9G, 2CltZ, DHZ7G, 2MeScZR, ACC, GA9, and SAG. MEcoral1 module had a high correlation with IAA-Ala, IAA-Glc, IAA-Glu, ICAld, KR, K9G, 2CltZ, DHZ7G, 2MeScZR, GA9, and SAG. As there were a few related hormone components in other modules, or the correlation pattern was similar to the above two modules, we selected MEhoneydew and MEcoral1 for further network model construction and analysis.

**Figure 8:**
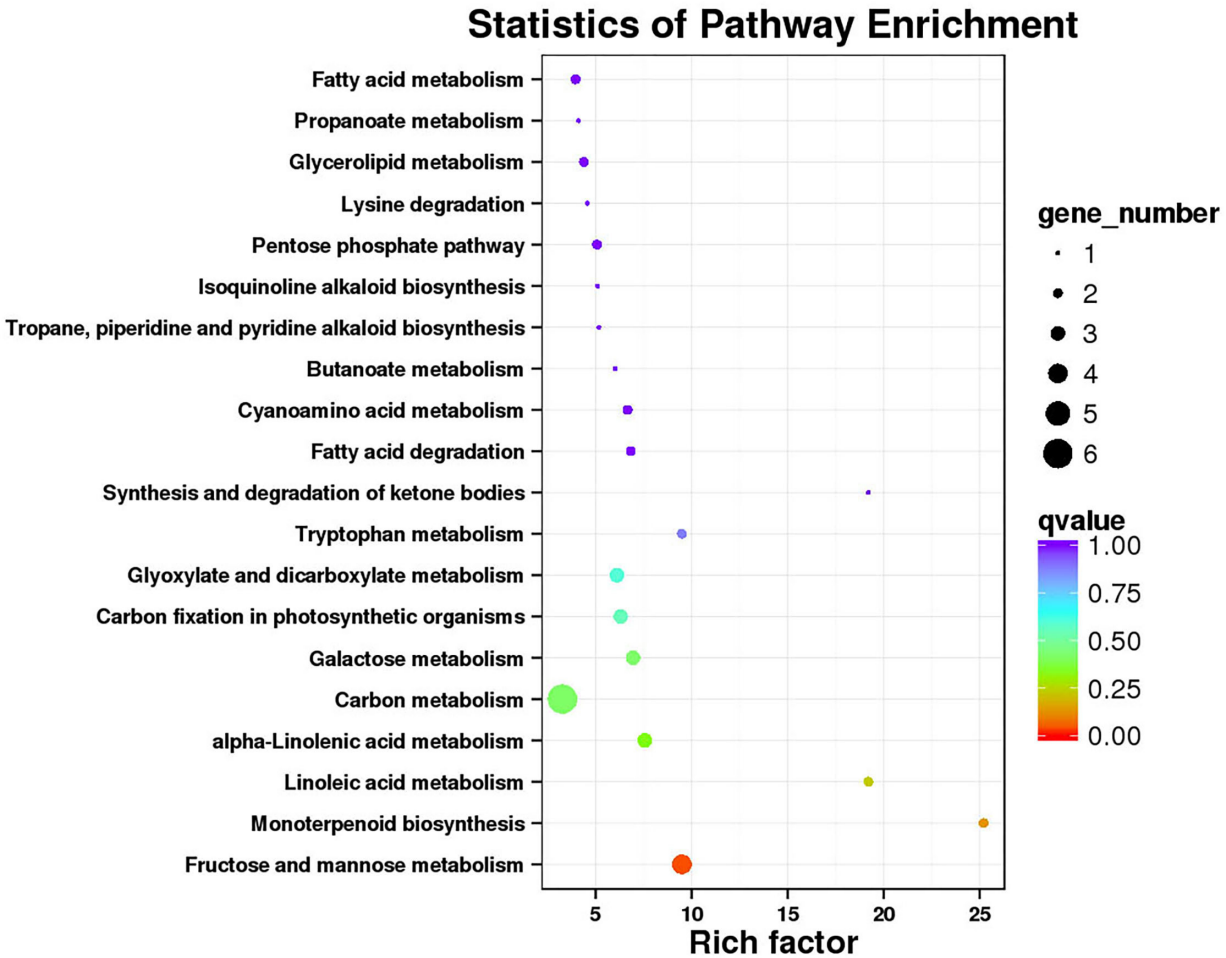
Functional annotation and Kyoto Encyclopedia of Genes and Genomes (KEGG) pathway enrichment of hub genes in MEhoneydew.

Functional annotation and enrichment of Kyoto Encyclopedia of Genes and Genomes (KEGG) genes were carried out on 150 core genes in the MEhoneydew module (Figure 8). Results showed that 8 genes were enriched in carbon metabolism-related pathways, namely, *ZB36497* (photosynthesis ko00195); *ZB07173, ZB18516*, and *ZB26838* (carbon fixation in photosynthetic organisms ko00710), *ZB07173, ZB18516, ZB19499, ZB20582, ZB26838*, and *ZB55699* (carbon metabolism ko01200); 7 genes were enriched in fatty acid metabolism pathways, namely, *ZB20582* and *ZB54028* (fatty acid metabolism ko01212), *ZB00998* and *ZB17366* (linoleic acid metabolism ko00591), *ZB20582* and *ZB54028* (fatty acid degradation ko00071), *ZB53339* (glycerophospholipid metabolism ko00564), *ZB00998, ZB17366*, and *ZB54082* (alpha-linolenic acid metabolism ko00592), *ZB16680* and *ZB30550* (glycerolipid metabolism ko00561); 8 genes were enriched in amino acid metabolism pathways, namely, *ZB20582* (lysine degradation ko00310), *ZB07173* and *ZB18516* (biosynthesis of amino acids ko01230), *ZB48222* (beta-alanine metabolism ko00410, glycine, serine, and threonine metabolism ko00260, tyrosine metabolism ko00350, phenylalanine metabolism ko00360), *ZB20582* (glycine, serine, and threonine metabolism ko00260), *ZB19499* and *ZB36273* (cyanoamino acid metabolism ko00460), *ZB20582* and *ZB55699* (tryptophan metabolism ko00380), *ZB39498* (cysteine and methionine metabolism ko00270); 12 genes were enriched in sugar metabolism pathways, namely, *ZB56404* (amino sugar and nucleotide sugar metabolism ko00520), *ZB07173* and *ZB18516* (glycolysis/gluconeogenesis ko00010), *ZB41084* and *ZB55060* (pentose and glucuronate interconversions ko00040), *ZB36689* (starch and sucrose metabolism ko00500), *ZB20582* and *ZB26838* (pyruvate metabolism ko00620), *ZB07173* and *ZB18516* (pentose phosphate pathway ko00030), *ZB07173, ZB18516, ZB41084*, and *ZB55060* (fructose and mannose metabolism ko00051), *ZB19499, ZB20582*, and *ZB55699* (glyoxylate and dicarboxylate metabolism ko00630), *ZB30111, ZB34843*, and *ZB56404* (galactose metabolism ko00052); 3 genes were enriched in signaling transduction pathways, namely, *ZB53339* (phosphatidylinositol signaling system ko04070 and inositol phosphate metabolism ko00562), *ZB43608* and *ZB56627* (plant hormone signal transduction ko04075).

**Figure 9:**
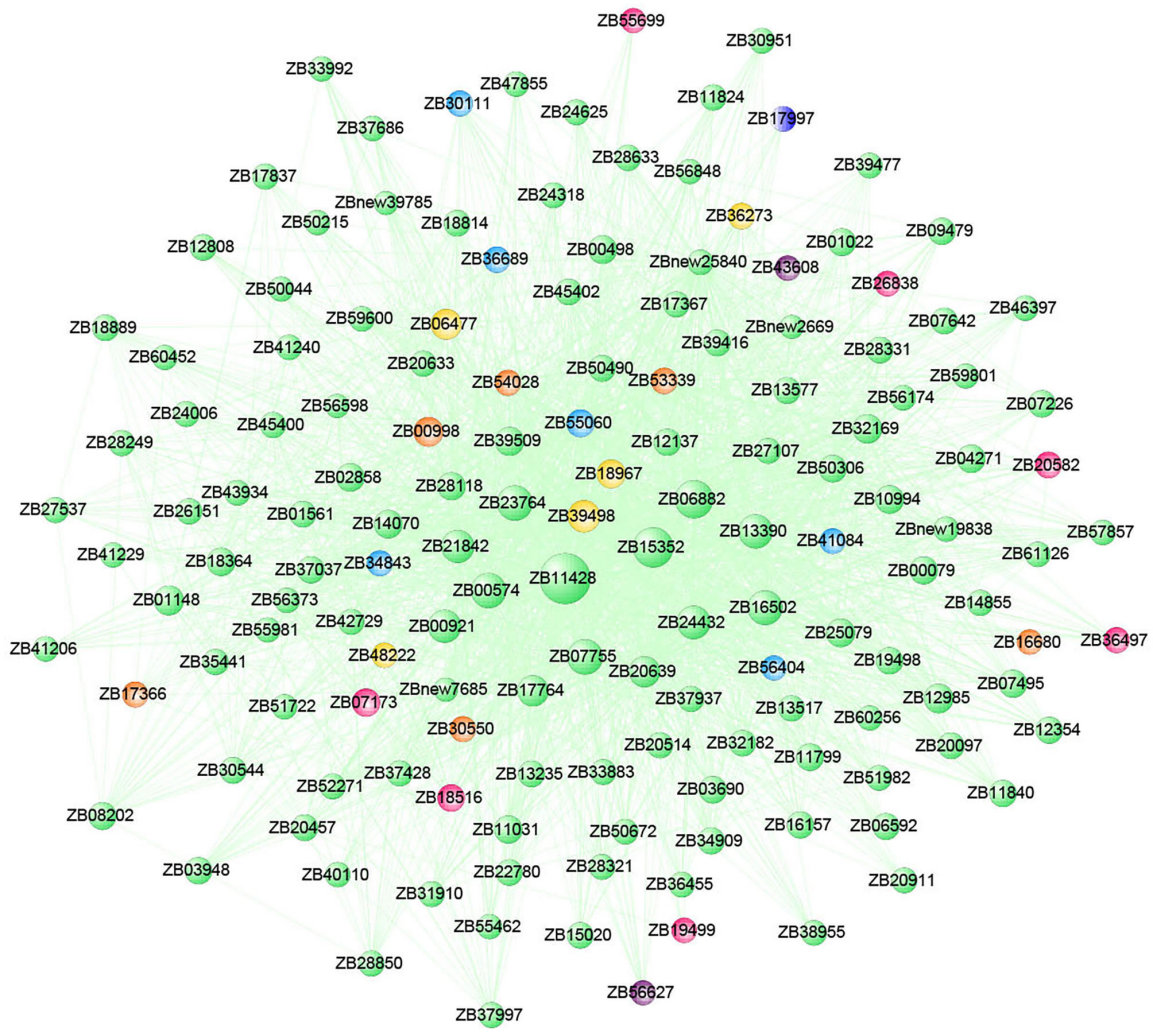
Co-expression network of the top 150 hub genes from the MEhoneydew module. The size of each gene node in the figure represents the connectivity of genes, and the node color represents the category.

The co-expression gene network was constructed with 150 hub genes in the MEhoneydew module, and the gene connectivity ranged from 0.10 to 17.93 (Figure 9). In this module, two hormone signaling pathway genes were identified. *ZB43608* was a TGA transcription factor in the salicylate hormone signaling pathway, which connected 14 genes in the network, namely, *ZB00574* (*RPS2*), *ZB06882, ZB11428, ZB15352* (*AL5*), *ZB16502* (*PUB5*), *ZB20639* (*RNP1*), *ZB23764* (*PUB14*), *ZB24432* (*GALK*), *ZB32169* (*AT4G26450*), *ZB39498, ZB39509* (*SRG1*), *ZB50490* (*CYP736A12*), *ZB55060* (*SDH*), and *ZBnew25840* (*Os03g0733400*). *ZB56627* was the two-component response regulator ARR11 in the cytokinine signal transduction pathway, which connected 12 genes in the network, namely, *ZB00574* (*RPS2*), *ZB07755, ZB11428, ZB15352* (*AL5*), *ZB17764* (*CNGC5*), *ZB21842, ZB23764* (*PUB14*), *ZB24432* (*GALK*), *ZB39498, ZB39509* (*SRG1*), *ZB55060* (*SDH*), and *ZBnew25840* (*Os03g0733400*). These genes were involved in spliceosome (ko03040), cysteine and methionine metabolism (ko00270), pentose and glucuronate interconversions (ko00040), and fructose and mannose metabolism (ko00051) pathways. Among them, *ZB11428, ZB15352, ZB06882, ZB07755, ZB23764, ZB00574, ZB16502*, and *ZB24432* had high connectivity in the module and were the key genes of the network.

**Figure 10:**
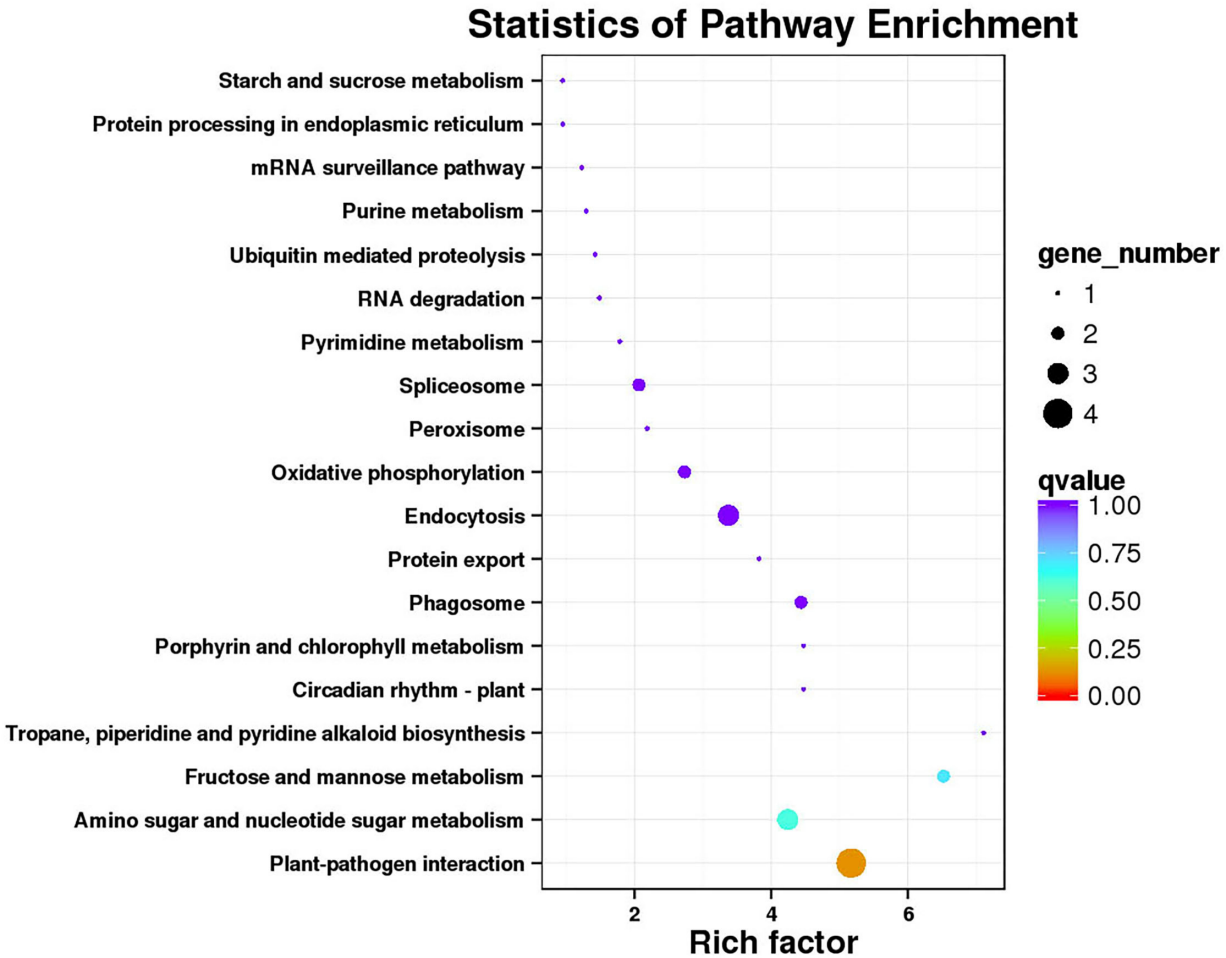
Functional annotation and KEGG pathway enrichment of hub genes in MEcoral1.

Functional annotation and enrichment of KEGG genes were carried out on 150 core genes in the MEcoral1 module (Figure 10). Results showed that 3 genes (*ZB36615* (starch and sucrose metabolism ko00500, amino sugar and nucleotide sugar metabolism ko00520), *ZB05065* and *ZB40450* [fructose and mannose metabolism ko00051, amino sugar and nucleotide sugar metabolism ko00520)] were enriched in the sugar metabolism pathways; 1 gene (*ZB29785*) enriched in the peroxisome ko04146 pathway; 3 genes (*ZB24496, ZB29882*, and *ZB54262*) enriched in the endocytosis ko04144 pathway; 1 gene (*ZB50206*) enriched in the tropane, piperidine, and pyridine alkaloid biosynthesis ko00960 pathway; 1 gene (*ZB48959*) enriched in the circadian rhythm-plant ko04712; 1 gene (*ZBnew19225*) enriched in the porphyrin and chlorophyll metabolism ko00860 pathway; and 2 genes (*ZB06550* and *ZB40291*) enriched in the phagosome ko04145 pathway.

**Figure 11:**
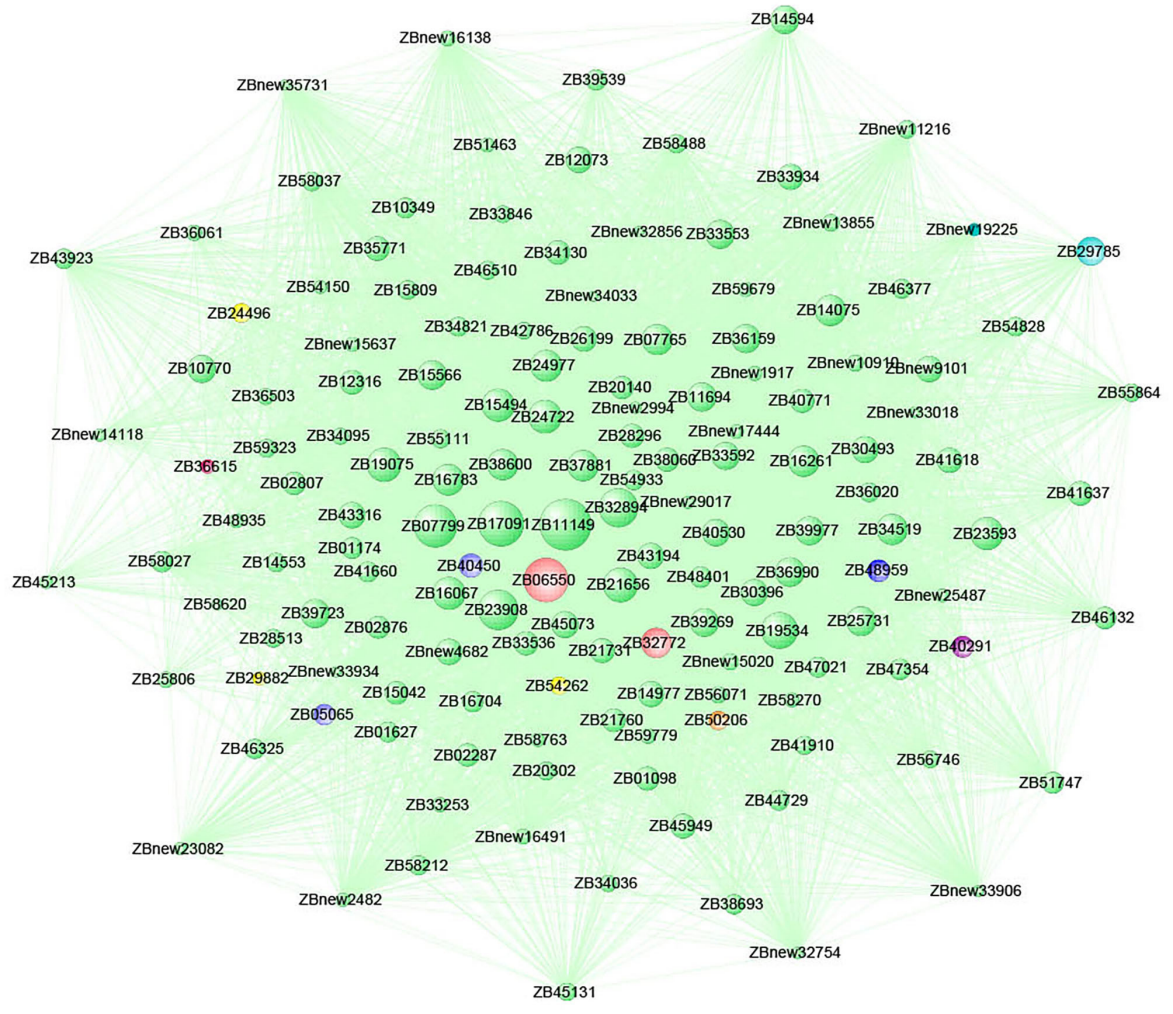
Co-expression network of the top 150 hub genes from the MEcoral1 module.

The gene connectivity in the MEcoral1 module co-expression network ranged from 0.11 to 18.92 (Figure 11). Among them, the gene *ZB11149* had the highest connectivity (c = 18.92) and participated in the intracellular protein transport, protein transporter activity and vesicle-mediated transport (GO: 0006886, GO: 0008565, and GO: 0016192) process; gene *ZB06550* (c = 15.59) participated in the vacuolar proton-transporting, plasma membrane, chloroplast, integral component of membrane (GO: 0000220, GO: 0005886, GO: 0009507, and GO: 0016021) process; gene *ZB32894* (c = 13.07) participated in calcium ion binding (GO: 0005509) and NAD(P) activity (GO: 0032440) process, which were related to signaling transduction; gene *ZB21656* (c = 11.15) was a ubiquitin-conjugating enzyme, which had acid-amino acid ligase activity; gene *ZB23593* was Bax1 inhibitor and participated in cellular component process (GO: 0016021). The above genes all had high connectivities and were in the core positions of the co-expression network.

## Discussion

Abscisic acid was considered to be an important hormone in plant response to low temperatures. As an important signal factor, ABA regulates plant cold response by regulating various stress pathways. One study showed that cold stress can increase the endogenous ABA levels in plants and then improve their resistance to low temperatures (Li et al., 2017). In our study, ABA was found to be the main hormone component in the two varieties of *Z. bungeanum*, indicating that ABA plays an important role in the cold response of *Z. bungeanum*. However, we found that the changing trend of ABA content varied in cold-tolerant and cold-sensitive varieties, indicating that the ABA response patterns of the two varieties were different. The level of ABA content in FG was significantly higher than that in FX at the early (0–1 h) and late stages (12–24 h) under cold stress. It has been reported that a large amount of ABA accumulates during low-temperature stress, and the content of ABA in cold-tolerant plants is higher than that in cold-sensitive plants (Lang et al., 1994; Wang et al., 2009). Under normal growth conditions, the content of ABA in FG was significantly higher than that in FX, which could play a faster role as a signal pathway in the early stage of low-temperature stress, so it made a more rapid response to cold. At the late stage (12–24 h) of low-temperature stress, the content of ABA in FG increased significantly. The level of endogenous ABA can increase temporarily in the initial stage of cold response, while continuous action requires transport and replenishment from other tissue parts (Shi and Yang, 2014). ABA-GE is one of the most abundant forms of ABA couplings. It showed very weak or almost no biological activity. For a long time, ABA-GE has been regarded as a by-product of catabolism that reduces the level of ABA in cells. Studies have shown that ABA-GE acts as an ABA reservoir and can be rapidly hydrolyzed to produce active ABA under the action of glucosidase (Johnson and Ferrell, 1982; Dietz et al., 2000; Sauter et al., 2002). The active ABA produced is very important for plants to adapt to abiotic stress. In addition, ABA-GE can supplement ABA by long-distance transport from the roots up to the leaves (Xu et al., 2014). In this study, we found that the ABA content in leaves of FG increased significantly at 12 and 24 h, while the content of ABA-GE also increased significantly at these time points. Sauter reported that the ABA-GE content increased significantly under stress, which was consistent with our results (Sauter et al., 2002). Moreover, OPLS-DA results showed that ABA-GE was an important differential hormone component in FG samples at 12–24 h. Therefore, we speculate that in the process of cold response in FG, ABA-GE acts as a storage and replenishment repository of active ABA and carries out cold response by long-distance transport from the roots to leaves at cold stimulation sites. This result also adds new evidence to the theory that ABA-GE can be transported over long distances in response to stress. In addition, transmembrane transporters are necessary for long-distance transport of ABA-GE, but the mechanism of it remains unclear. In the WGCNA co-expression module MEcoral1, the gene *ZB1119* with the highest connectivity was annotated to participate in intracellular and vesicle transport, so it was speculated that it may be related to the transmembrane transport of plant hormone. Further experiments and evidence are needed to confirm this.

Cytokinin is the central regulator of plant development, which can promote cell division and participate in biological processes such as plant growth and flowering, leaf senescence, and chloroplast generation (Khan et al., 2017). The effect of low-temperature stress on plants is the opposite. Therefore, a stable level of cytokinin plays a crucial role in the normal growth and development of plants under low temperatures. There is a wide variety of cytokinins in plants, including free state and binding state. It is generally believed that the cytokinin binding state form is more stable and that the two state forms can be converted to each other by the action of related enzymes (Zhang et al., 2003). In our results, cytokinin binding forms, such as cZROG, iP9G, DHZ7G, and K9G, were found to be important components in the low-temperature response pathway of CK in *Z. bungeanum*. It is suggested that the translational replenishment of binding form is the main way of cytokinin pathway regulation in *Z. bungeanum*. In addition, the changes in hormone content revealed that the response time and pattern of these binding state cytokinins differed in cold-tolerant and cold-sensitive varieties. For example, the levels of cZROG and DHZ7G were upregulated in FG, while downregulated in FX. This also provides a basis for the follow-up study on the differential exogenous hormone application between the two varieties.

Auxin plays an important role in the whole plant growth cycle, mainly in regulating cell elongation, organ morphogenesis, and interacting with other hormones. One of the most important characteristics of auxin is polar transport, which mostly depends on the polar distribution of PIN family proteins on the cell membrane (Geldner et al., 2001). One study found that endocytosis and exocytosis in cell activities were involved in the polar distribution of PIN protein on the cell membrane (Dhonukshe et al., 2007). In our previous study, we found that endocytosis played an important role in the cold response network of *Z. bungeanum*, and the endocytosis-related gene *ERD7* was a key gene in the co-expression network, with the highest connection with other genes and located in the center of the network (Tian et al., 2021). In this study, the MEcoral1 module also identified 3 genes (*ZB24496, ZB29882*, and *ZB54262*) enriched in the endocytosis (ko04144) pathway. Moreover, TRP, an important initiator in the IAA synthesis pathway, was found to be the most abundant of all hormones in the two varieties of *Z. bungeanum*. These results suggested that the polarity distribution of auxin mediated by endocytosis is an important way and action form of *Z. bungeanum* response to low-temperature stress. In the IAA biosynthesis pathway in *Z. bungeanum*, we also found that ICAld and ICA were the important upregulated hormone components. A study reported that the concentration of ICA in the cell wall increased significantly under pathogen attack and that indolic derivatives may serve as structural scaffolds for cell wall modifications after the attack (Forcat et al., 2010). The plant cell wall is the first barrier against low temperature. Therefore, we speculated that ICA has the same cell wall scaffolding role in *Z. bungeanum* under cold stress.

The common hormone components, such as ABA, ABA-GE, ICAld, and TRP, were found in the two varieties of *Z. bungeanum*, and the hormone components specifically accumulated in each variety, such as IA, IAA-Glu, and GA9 in FG and IAA-Glc in FX, were also found. This result indicated that the hormone response system of *Z. bungeanum* under low-temperature stress was complex and diverse, and the response patterns varied in cold-tolerant and cold-sensitive varieties. Plant hormone interactions under abiotic stresses are also receiving increasing attention. Strigolactones were found to alleviate low-temperature injury in tomatoes (*Solanum lycopersicum*) through the ABA pathway (Chi et al., 2021). Another study found that ABI5, a transcription factor of the ABA signaling pathway, was involved in regulating the auxin, CK, GA, and IAA hormone signaling pathways in transgenic potatoes (Li, 2019). Various hormones in plants show a dynamic network of physiological effects that promote and antagonize each other, and the dynamic balance between these hormones is very important for maintaining normal growth and development under adversity. The content levels and change patterns of each hormone component obtained in this study provide a basis for subsequent studies of crosstalk among hormones under low-temperature stress.

The CHM and UMAP analysis of the samples treated with low temperature showed that the samples of 0 and 1 h, 3 and 6 h, and 12 and 24 h in FG were clustered into a group, respectively, indicating that the hormone components and levels of FG were adjusted correspondingly under different time periods of cold stress. NMDS analysis showed that the hormone components in the samples in FX at 6 h were close to the samples in FG at 12 and 24 h, indicating that the hormone response to low-temperature stress was the strongest at 6 h of FX and the ability to resist cold was at a high level. OPLS-DA analysis results showed that in the cold response process of FG and FX, there were not only important differential hormone components with the same expression pattern but also hormone components with the opposite expression pattern. This indicated that with the extension of cold stress treatment time, there were common crossing points and independent response pathways in the two varieties of *Z. bungeanum* under cold stress.

In addition, hormone-related gene modules were constructed by WGCNA, among which the MEhoneydew and MEcoral1 modules were highly correlated with the main hormone components. The response pathway of hormone components in *Z. bungeanum* under low-temperature stress was related to both upstream and downstream metabolic pathways, including carbon, fatty acid, amino acid, and sugar metabolism. These results indicate that hormone regulation plays an important role in the response of *Z. bungeanum* to low temperatures. Moreover, the identified hub genes are located in the center of the hormone response network pathway, and their regulatory effects are more sensitive and faster, which can be used as candidate genes for further study of low-temperature response.

## Conclusion

The main aim of this study was to investigate the variation in hormone component profiling in *Z. bungeanum* under low-temperature treatment. In the leaves of two *Z. bungeanum* varieties, 45 main plant hormone components were detected, and ABA, ABA-GE, ICAld, TRP, ACC, SA, and SAG were the main high-level content of hormones. Low temperature affected the components and contents of plant hormones in *Z. bungeanum*, and the cold response varied in the two varieties. In the low-temperature response process of FG, ABA-GE can serve as the storage and replenishment repository of active ABA and respond to low temperature by long-distance transport from root tissue to leaves at the low-temperature stimulation site, but the same result was not found in FX. The polarity distribution of auxin mediated by endocytosis may be an important way and form of *Z. bungeanum* response to cold stress. OPLS-DA results showed that the important differential hormone components vary in FG and FX, and there were intersections and independent response pathways of plant hormones. WGCNA analysis showed that hormones, as upstream signals of low-temperature stress, were associated with other response pathways in plants, including carbon, fatty acid, sugar, amino acid metabolism, and other signal transduction pathways. Two hormone-related core genes *ZB43608* (a TGA transcription factor in the salicylate hormone signaling pathway) and *ZB56627* (a two-component response regulator ARR11 in the cytokinin signaling pathway) were identified. The results of this study revealed the hormone response strategy of *Z. bungeanum* under low-temperature stress in terms of composition and content level and provided a further reference for improving the cold resistance of *Z. bungeanum* and breeding-resistant varieties.

## Data Availability Statement

The datasets presented in this study can be found in online repositories. The names of the repository/repositories and accession number(s) can be found below: https://www.ncbi.nlm.nih.gov/, PRJNA597398.

## Author Contributions

AW and JT conceived and designed the research. JT performed experiments, analyzed the results, and wrote the manuscript. YM revised the manuscript. YC and XC performed some experiments. All authors reviewed and approved the manuscript.

## Funding

This study was supported by the Shaanxi Province Technical Innovation Guidance Project Funding (Grant No. 2020QFY07-01).

## Conflict of Interest

The authors declare that the research was conducted in the absence of any commercial or financial relationships that could be construed as a potential conflict of interest.

## Publisher's Note

All claims expressed in this article are solely those of the authors and do not necessarily represent those of their affiliated organizations, or those of the publisher, the editors and the reviewers. Any product that may be evaluated in this article, or claim that may be made by its manufacturer, is not guaranteed or endorsed by the publisher.
